# C-Type Lectins in Veterinary Species: Recent Advancements and Applications

**DOI:** 10.3390/ijms21145122

**Published:** 2020-07-20

**Authors:** Dimitri Leonid Lindenwald, Bernd Lepenies

**Affiliations:** Immunology Unit & Research Center for Emerging Infections and Zoonoses (RIZ), University for Veterinary Medicine Hannover, Foundation, 30559 Hannover, Germany; Dimitri.Leonid.Lindenwald@tiho-hannover.de

**Keywords:** C-type lectin, glycans, immune modulation, comparative immunology, veterinary immunology

## Abstract

C-type lectins (CTLs), a superfamily of glycan-binding receptors, play a pivotal role in the host defense against pathogens and the maintenance of immune homeostasis of higher animals and humans. CTLs in innate immunity serve as pattern recognition receptors and often bind to glycan structures in damage- and pathogen-associated molecular patterns. While CTLs are found throughout the whole animal kingdom, their ligand specificities and downstream signaling have mainly been studied in humans and in model organisms such as mice. In this review, recent advancements in CTL research in veterinary species as well as potential applications of CTL targeting in veterinary medicine are outlined.

## 1. Introduction

**Figure 1:**
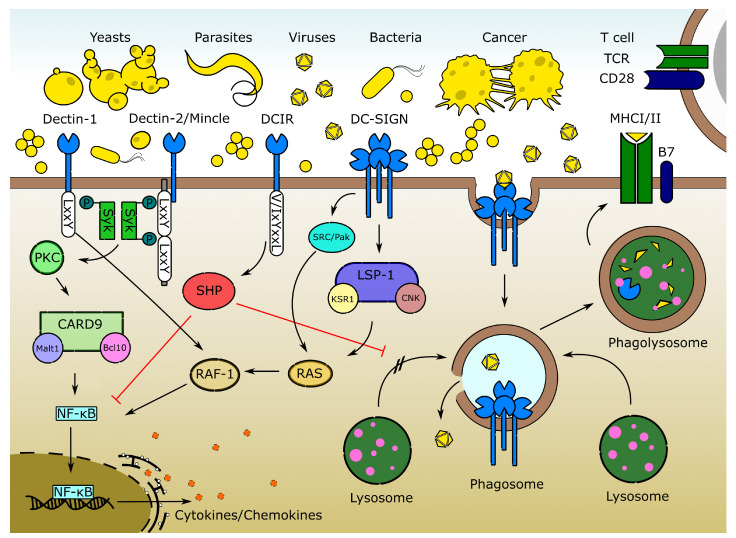
CTL functions and signalling pathways. CTLs recognize molecular patterns of fungal, parasitic, bacterial, and viral pathogens (so-called PAMPs) as well as those of dead and malignant cells (DAMPs). Upon pathogen binding, CTL–mediated signalling leads to cytokine and chemokine production and phagocytosis. The latter results in antigen (cross-)presentation and priming of T-cells. However, some viruses, such as the zoonotic Dengue fever virus, developed immune evasion mechanisms and may exploit CTLs such as DC-SIGN to promote viral transmission and dissemination.

Glycans belong to the most abundant macromolecules constituting all living organisms. In multicellular animals, processes such as cell migration, homeostasis maintenance, and innate immune signaling rely on the ability of cells to recognize glycoconjugates, most often in the form of glycoproteins and glycolipids, via glycan binding proteins, the so-called lectins [1]. In the immune system, lectin receptors are either secreted or found on the cell surface of immune cells [2]. Three major receptor families that are involved in glycan recognition in the immune system include the galectins [3], siglecs [4], and C-type lectins (CTLs) [5]. Among these, the phylogenetically conserved CTLs proved to play a pivotal role in both host–pathogen interactions and homeostasis maintenance in vertebrate and in invertebrate species [5,6,7]. Myeloid CTLs are mainly expressed by antigen-presenting cells (APCs) and act as pattern recognition receptors (PRRs) that bind to pathogen and damage-associated molecular patterns (PAMPs and DAMPs) [5]. Most CTL receptors require Ca^2+^ ions for binding, hence the “C” in the name. However, some CTLs also bind carbohydrate, peptide, or crystalline ligands in a Ca^2+^-independent manner [5]. The importance of CTLs for antifungal immunity is well recognized in human medicine [8] (Table 1). For instance, an increased risk for candidiasis [9] and a higher susceptibility to aspergillosis is associated with CTL polymorphisms in human patients [10]. However, CTLs are also chiefly important in the scope of immune homeostasis [11,12,13,14] and protection against bacteria, viruses, parasites, and cancer [15,16,17,18,19] (Figure 1). They induce signal pathways leading to the expression of chemokines and cytokines, and they are involved in phagocytosis and antigen (cross-)presentation by molecules of the major histocompatibility complex (MHC) I or II to T-cells, thus bridging innate and adaptive immunity [20] (Figure 1). CTLs associated with an immunoreceptor tyrosine-based activation motif (ITAM), such as the dendritic cell-associated lectin 1 (Dectin-1/Clec7a), and 2 (Dectin-2/Clec6a) and the macrophage-inducible Ca^2+^-dependent lectin (Mincle/Clec4e), signal upon ligand binding via phosphorylation of the spleen tyrosine kinase (Syk). Syk activates further kinases such as the protein kinase C (PKC), which results in downstream activation and assembly of the caspase recruitment domain-containing protein 9 (CARD9), mucosa-associated lymphoid tissue lymphoma translocation protein 1 (MALT1), and B-cell lymphoma protein 10 (BCL10) complex. Finally, this leads to phosphorylation of IκB and translocation of the transcription factor NF-κB into the nucleus, where it enhances the transcription of numerous cytokine and chemokine genes [21]. This activation may be counteracted by CTLs such as the DC immunoreceptor (DCIR/Clec4a), which carry an immunoreceptor tyrosine-based inhibition motif (ITIM) and engage the src homology domain-containing protein tyrosine phosphatases (SHP), thus restricting ITAM-mediated signals and limiting inflammation [22,23]. ITAM/ITIM-independent CTLs, such as the dendritic cell-specific ICAM-3-grabbing non-integrin (DC-SIGN/Clec4l/CD209) can also stimulate the activation of NF-κB via steroid receptor coactivator (SRC) and p21-activated kinase (PAK) or via the leukocyte-specific protein 1 (LSP-1), kinase suppressor of RAS 1 (KSR-1), and connector enhancer of kinase suppressor of RAS (CNK) rat sarcoma (RAS) signalosome [22]. However, CTLs were also shown to act as pathogen entry receptors and targets of immune escape [24] and may contribute to immune pathology in several infections [25,26,27,28], as well as in autoimmune diseases and cancer [20,29,30].

**Figure 2:**
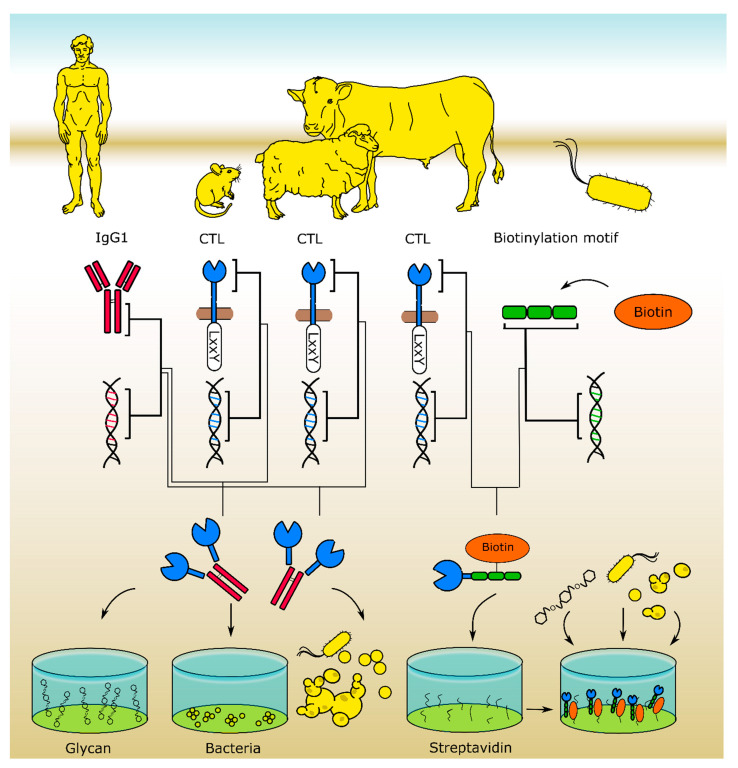
Recombinant CTL libraries for in vitro screenings allow for the identification of CTL ligands. The murine [31] and ovine [60] CTL libraries were expressed as CTL-Fc fusion proteins. For the bovine [61] library, cow CTL and bacterial biotinylation site coding DNA fragments were fused and expressed in *E. coli*, yielding biotinylated fusion proteins that can be used for glycan array- and ELISA-based binding studies and high throughput pull-down assays.

**Figure 3:**
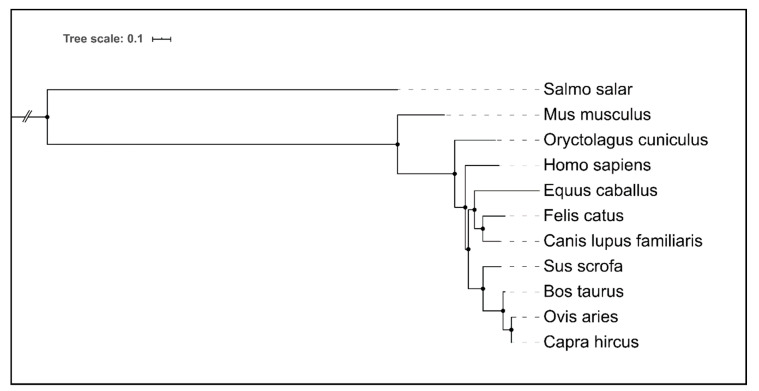
Hierarchical clustering of amino acid sequences comprising Dectin-1 (Clec7a) CTLs of selected animal species, and humans. For the Atlantic salmon (*Salmo salar*), missing a corresponding ortholog Dectin-1 encoding gene, a functional ortholog C-type lectin receptor C was chosen. Remarkably, the degree of similarity in the Dectin-1 amino acid sequences mirrors the phylogenetic relationships between the respective species. Visualization and clustering were performed with NGPhylogeny.fr suite [62].

Most insights into animal CTLs functions were gained in studies performed with model organisms, predominantly mice. In vitro CTL–ligand screenings using murine [31,32,33] or human [34,35] recombinant CTL hFc-fusion protein libraries (Figure 2) allowed for the identification of novel CTL/pathogen interactions and CTL ligands [34,36]. Further studies analyzed ligand binding and downstream signal transduction of mouse and human CTL using APCs [21,37,38,39,40,41] or CTL expressing reporter cell systems [42,43,44,45]. Data from human patients [46] and studies performed in CTL^−/−^ mice or mice that were deficient for CTL-mediated signaling [47,48] depict the effects of particular CTLs in vivo. However, ligand specificities of CTL orthologues, downstream signaling pathways, and effector functions may significantly vary among different species [44,49,50,51,52,53,54,55,56,57,58,59], thus emphasizing the need for CTL investigations performed in a species-specific manner (Figure 3). In particular, there is a knowledge gap regarding CTL function in veterinary species. In the following sections, we will discuss recent studies in this field and briefly highlight potential applications of CTL targeting in veterinary medicine.

## 2. Protective Role of Veterinary Relevant CTLs

Most often, CTL functions in veterinary species were investigated in population screening studies. These studies correlated the course of infection- or general susceptibility-associated phenotypes with specific CTL genotypes, thus allowing for conclusions concerning the functions of individual CTLs in health and disease [63,64]. By using such strategies, implications of CTLs in antimicrobial immunity were recently described. For instance, a link between different single nucleotide polymorphisms (SNPs) in the bovine Dectin-1 encoding gene and the susceptibility to Johne’s disease caused by *Mycobacterium avium* ssp. *paratuberculosis* (MAP) was found in screening studies in Canadian [65] and in Indian cattle [66]. Similarly, multiple SNPs were described in the Dectin-1 encoding gene in pigs [67]. All SNPs discovered in commercial pig lines proved to be neutral when compared to the reference pig Dectin-1 in a NF-κB driven reporter system using the Dectin-1 ligand zymosan [67]. In contrast, the Dectin-1 isoform found exclusively in wild boars displayed a markedly enhanced activatory capability upon ligand stimulation. This augmented Dectin-1 signaling was suggested to negatively influence the overall fitness of its carriers, possibly leading to overshooting immune responses to pathogenic and commensal fungi [67]. A SNP in the gene encoding for the humoral mannose binding lectin A (MBL1), on the other hand, was hypothesized to result in a loss-of-function type of mutation and in an increased shedding of *Salmonella* sp. in fattened pigs [68]. Consistently, a negative correlation between the concentration of the orthologous MBL and *Salmonella* susceptibility was observed in chicken [69]. However, multiple SNPs in the non-coding intron parts of the MBL gene were shown to correlate with varying serum MBL levels in Chinese Hu sheep [70], demonstrating that SNPs that do not directly affect the CTL protein sequence may nevertheless influence CTL levels in vivo.

The antimicrobial effects of several CTLs were recently shown for both sweet water [71] and salt water [72] fish species. In carp, a number of CTLs were identified to be downregulated on macrophages upon stimulation with the ß-glucan curdlan [73], which is a well-known ligand of Dectin-1 in mammals [74]. This surprising effect may represent a negative feedback mechanism preventing an over-stimulation of the carp immune cells in the course of bacterial and fungal infections [73]. In contrast, several salmon genes encoding signaling molecules downstream of CTL receptors SCRLA, SCRLB, and SCRLC (Salmon C-type lectins A,B,C), such as the one encoding the fish analogue of the mammalian tyrosine kinase Syk, were significantly upregulated following ß-glucan stimulation. These findings suggest an involvement of CTLs in pathogen recognition and signal transduction in salmon [75].

A strong correlation between the protective Th1 response and Dectin-1 engagement was recently shown in mouse *Leishmania* spp. infections models [76,77], demonstrating a crucial role of this specific CTL in anti-*Leishmania* immunity. The site-specific expansion of Dectin-1 expressing DCs following intradermal injection of the specific Dectin-1 agonist curdlan sufficed to protect wild-type mice from illness following transdermal *Leishmania* infection, whereas Dectin-1^−/−^ mice succumbed to the disease [76]. Leishmaniosis is an important and life-threatening disease in dogs; an insufficient Th1 response in favor of the detrimental Th2 response [78] in clinically affected canids renders vaccine development a significant challenge [79,80]. However, the function of canine Dectin-1 during *Leishmania* spp. infection in dogs is yet unknown.

The influence of different CTL-associated alleles on anti-parasitic immunity was described in wild Soay sheep on the St Kilda archipelago, Scotland [81]. In this study, SNPs in the presumed cis-regulatory element of the *clec16a* gene, a CTL-encoding gene associated with immunoglobulin isotype deficiency disorders in humans and mice [82], strongly correlated with specific IgA levels against the intestinal roundworm *Telodorsagia circumcincta* in lambs as well as in mature sheep [81]. In fish, CTLs may also contribute to protective immune responses against parasites as suggested by a positive correlation between macrophage mannose receptor 1 (MRC1/Clec13d) expression levels and the relative resistance of Atlantic [83] and pink salmon toward sea lice infestation [84]. These findings indicate that the selective breeding or genetic engineering introducing desirable CTL alleles into veterinary species might be a means to improve their performance and disease resistance in the future.

## 3. Detrimental Role of Veterinary Relevant CTLs

### 3.1. Pathological Inflammation

Dysregulation in CTL signaling can lead to sterile inflammation in the absence of any pathogen [29]. For instance, a possible involvement of Dectin-1 in sterile inflammation and postpartum placenta retention was suggested in cows, since higher numbers of Dectin-1-expressing uterine macrophages were detected in retention-affected cows compared to cows with a regular afterbirth [85]. Allergic hypersensitivity and immunopathology can also be mediated by CTLs: in horses, Dectin-1, Dectin-2, and macrophage lectin 2 (MGL/Clec10a) may contribute to severe allergic dermatitis following insect bites [86]. Similar findings were also obtained for mice and men, as Dectin-1^−/−^ mice were largely protected against *Aspergillus fumigatus*-initiated corneal keratitis [87] and Dectin-1 blockade using the antagonist laminarin alleviated the severity of fungal keratitis in human patients [28]. Other CTLs may also be involved in immune pathology upon CTL engagement during infections. For instance, the myeloid C-type lectin-like receptor (MICL/Clec12a) was shown to cross-prime CD8^+^ T-cells contributing to the development of experimental cerebral malaria [26] and to promote murine viral lymphocytic choriomeningitis virus (LCMV) infections by hampering pathogen clearance [88]. To date, there is a knowledge gap on how CTLs may contribute to immune pathology in veterinary species, thus highlighting the need for further research in this field.

### 3.2. Exploitation of CTLs by Pathogens

Numerous viral pathogens, among them arthropod-borne phleboviruses, such as Dengue virus and Rift Valley Fever virus, specifically target CTLs such as the human DC-SIGN to establish infections [89]. Similarly, the feline corona virus, a close relative of both the canine coronaviruses (CCoVs) and the porcine transmissible gastroenteritis virus (TGEV) [90], establish infection by exploiting the cat DC-SIGN [91]. Heterologous expression of human DC-SIGN in otherwise resistant cells rendered them susceptible for infection with an avian corona virus, chicken Infectious Bronchitis virus (IBV) [92]. These studies on viral/DC-SIGN interactions indicate that CTLs represent relevant receptors for viral entry into host cells; thus, they may play a crucial role in the cross-species transmission of viruses.

However, not only viruses, but also bacteria and parasites were reported to highjack DC-SIGN or its orthologues, as recently demonstrated for the bacterium *Yersinia pestis* [93] and the apicomplexan parasite *Toxoplasma gondii* [94]. For the ruminant trematode parasite *Fasciola hepatica*, a strong downregulation of host DC effector functions via DC-SIGN was observed, finally leading to immune dysregulation and T-cell anergy [95]. The apicomplexan parasite *Neospora caninum* circulating between canine definitive hosts and bovine intermediate hosts causes large losses in dairy and beef production worldwide by inducing abortions [96]. In a murine model, *N. caninum* engaged Dectin-1 and thereby inhibited DC effector functions in wild-type mice compared to Dectin-1^−/−^ mice [97]. Further helminths, such as the nematode *Toxocara canis*, were shown to synthesize a repertoire of mammalian-like CTLs [98,99] and unusual glycans [100], which might interfere with and subvert CTL-based glycan recognition in vivo [100]. In conclusion, the examples highlighted here demonstrate a variety of immune evasion strategies of parasites to interfere with mammalian CTL-mediated immunity [101].

## 4. Harnessing the Power of CTLs

CTLs represent attractive targets for immune modulation, not only in mice and humans, but also in veterinary species as they hold promise of novel and/or improved diagnostic, prophylactic, and therapeutic applications. In the following, we will briefly highlight some recent examples.

### 4.1. General Aspects

In veterinary research, cell-surface expressed CTLs can be used as cell-specific markers, thus allowing for the discrimination of immune cell subsets to elucidate specific functions. For instance, the analysis of antiviral effector functions of porcine DCs was performed in Clec13B/LY75/CD205 positive DCs [102]. Accordingly, the in vitro characterization of rainbow trout immune cell subpopulations was performed by staining Clec4t1 positive monocyte-derived macrophage and DC precursor cells with CTL-specific antibodies [71]. Plate-bound or soluble recombinant CTL-based fusion proteins can in turn be used for binding studies with bacteria, viruses, fungi, and parasites in order to identify interactions of CTLs with PAMPs [103]. In this regard, the lectin array technology offers excellent opportunities for the diagnosis of blood and urine infections [61] or protein glycosylation-associated disorders [104] in animal or human patients.

### 4.2. Prophylaxis

CTL-targeting adjuvants, for instance the Mincle glycolipid ligand trehalose-6,6-dimycolate (TDM) derived from the *M. tuberculosis* cell wall and its synthetic analogue trehelose-6,6-dibehenate (TDB) [105], were recently evaluated for their immunogenic properties in mouse models [106,107]. In veterinary research, this approach was adopted for the development of a bovine tuberculosis vaccine [108]. In addition, the co-application of TDB and furfurman (targeting Dectin-2) in pigs as well as TDB and curdlan (targeting Dectin-1) along with further PRR targeting ligands in cattle markedly enhanced vaccination efficacy against Foot-and-Mouth Disease by providing robust and long-lasting effects in vivo [53]. Similarly, a TDB-based experimental liposomal vaccine adjuvant, CAF01, was shown to mediate long-lived *M. tuberculosis*-specific T-cell responses in humans [109] and enhanced the efficacy of a commercially available inactivated influenza vaccine in ferret models [110]. This adjuvant system was also used for rainbow trout immunization against *Aeromonas salmonicida* and induced enhanced cellular immunity in comparison to formulation with the standard adjuvant mineral oil [111]. Further trehalose-based CTL-targeting compounds, such as the 2-hydroxy benzoic acid coupled trehalose compound 6,6′-bis-(3,5-di-tert-butylsalisate)-α,α-trehalose (UM1024), demonstrated high Mincle targeting specificity and low cytotoxicity in mouse and human peripheral blood mononuclear cells (PBMCs) in vitro and robust immunogenicity in a mouse model [112]. In pigs, a recently characterized MICL ortholog was proposed as a selective antigen delivery target, since it mediated antigen uptake by pig dendritic cells in vitro [113]. In poultry, novel CTL-targeting compounds such as pustulan [114], as well as other promising CTL immunization targets, namely Clec13B [115,116], Clec17AL-A, and –B [117], the bird homologues of the mammalian Prolectin/Clec17A, were described. These studies indicate that CTLs in veterinary species are indeed promising targets to enhance vaccine efficacy; however, further research is needed to evaluate the potential of the respective adjuvant candidates in vivo.

### 4.3. Therapeutic Applications

CTL targeting can not only enhance the efficacy of vaccines, but it can also be adapted to metaphylaxis and therapy. As a potential treatment of parasitic infections, T-cell modulation toward the favorable T helper type 1 response was achieved via the metaphylactic curdlan stimulation of Dectin-1 expressing DCs in the mouse model of cutaneous leishmaniosis. In this model, the co-injection of curdlan along with infectious *L. major* promastigotes resulted in a resistant phenotype observed in the otherwise highly susceptible BALB/c mouse strain [76]. This finding matches the protective properties of yeast glucans that had previously been described in murine leishmaniosis models [118,119]. The utility of this approach for leishmaniosis treatment in other species, such as the domestic and feral dogs [120], cats [121], and foxes [122] remains to be investigated.

DC-SIGN can serve as an adhesion and dissemination receptor for cat-born *Toxoplasma gondii* infection [94]. Therefore, the specific antibody- or glycan-mediated blocking of paralogues could possibly be applied to prevent toxoplasmosis in chicken [123], thus reducing the risk of alimentary infections in poultry meat consumers. Additionally, selective blocking of the corresponding DC-SIGN orthologue might be applied to limit IBV spread in chickens. Although no chicken DC-SIGN orthologue has been identified yet [92], it is probable to exist due to the engagement of human DC-SIGN and the DC-SIGN-related protein L-SIGN (CD209L/Clec4m) by IBV to establish experimental infections in vitro [92].

Furthermore, CTLs represent important therapeutic targets in immune-mediated diseases. As such, isolated helminth immunomodulatory compounds mimicking an infestation might be used to suppress autoimmunity in human patients [124]. Consistently, a desensitizing DC targeting construct composed of the mite allergoid and mannan, a ligand of the murine [125], ovine [60], and human [126] CTL Dectin-2, was described as a potential allergy treatment in dogs [127]. Another potential therapeutic CTL target is Mincle. The expression of Mincle along with Syk and CARD9 adapter proteins was described in cattle papillomavirus-associated urothelial tumor cells, suggesting their phagocytotic capacity and rendering Mincle a promising target in veterinary oncology [128].

## 5. Conclusions

Along with other PRRs, CTLs also are important constituents of the host–microbiome communication interface: symbiotic microbes interact with CTLs [129] and affect host cytokine production and CTL expression in trained innate immunity [130] and homeostasis [131] by epigenetic mechanisms. For the Dectin-1 targeting mushroom glucans lentinan [132] and proteo-β-glucan [133], a robust Dectin-1-mediated antidepressant-like effect was demonstrated in mouse models [133,134], illustrating the influence that CTLs may have upon animal and human cerebral functions via the microbiota–gut–brain signaling axis [135]. Many veterinary and human nutraceuticals, or pharmacologically active nutrition additives [136,137], are also likely to exhibit their respective immune stimulating and/or modulatory functions via CTL-mediated signalling. Such an effect was also observed in a study performed in crayfish: crayfish susceptibility to the viral White-spot disease was reduced while the expression of hemocyte-associated crayfish CTL (X2C306-1) was simultaneously upregulated following the probiotic gavage of *Bacillus amyloliquefaciens* [138]. Further positive effects of carbohydrate supplements were demonstrated in lentinan-rich shiitake mushrooms gavage in a rat model of human dyslipidemia [139], probiotic glucan gavage in carp [140], and mannoprotein supplementation in adult and aging dogs [141].

Finally, functions of CTLs in intrauterine immunity and maternal–fetal tolerance [142], as well as in parturition [143], were shown in humans. Initial studies suggest a possible involvement of CTLs in veterinary species in these processes. For instance, a microarray-based differential gene expression investigation in pregnant sheep yielded several candidate CTLs, such as the DCAR/Clec4b, that were upregulated during the early gestation phase in the endometrium [144]. However, the impact of the respective CTLs on the placenta immunity in vivo is an open question for future research.

In conclusion, advancements in the understanding of CTL functions in veterinary species will open up new applications in veterinary medicine; yet, the current lack of knowledge clearly highlights the need for further research. To bridge this knowledge gap between model and target species, novel tools, such as recombinant bovine [61] and ovine [60] CTL receptor libraries, were recently generated. The role of the identified CTL interactions of veterinary relevant species with pathogens will be unravelled in further studies.

## Figures and Tables

**Table 1:** Overview of selected human CTLs, including examples of respective ligands and functions.

C-Type Lectin	Main Expression	Ligands	Recognized Pathogens (Examples)
Dectin-1	Monocytes, Macrophages, Dendritic cells, NK cells,	(1→3)-β-D-glucans	*C. albicans*, *A. fumigatus*, *C. neoformans*, *Mycobacterium* spp.
Dectin-2	Monocytes, Macrophages, Dendritic cells, NK cells, Endothelial cells,	high-mannose oligosaccharides	*C. albicans*, *A. fumigatus*, *M. tuberculosis*, *S. mansoni*
Mincle	Monocytes, Macrophages, Dendritic cells,	mycobacterial trehalose 6,6’-dimycolate (TDM), alpha-mannose residues, DAMPs	*Mycobacterium* spp., *Malassezia* spp.
DC-SIGN	Dendritic cells	high-mannose and fucose-containingoligosaccharides	HIV-1, Dengue virus, Measles virus, SARS coronavirus
DCIR	Monocytes, Macrophages	Mannose, fucose	HIV-1
MICL	Macrophages, Monocytes, Granulocytes,	DAMPs, urate crystals, hemozoin	Unknown
MGL	Monocytes, Dendritic cells	terminal galactose and *N*-acetylgalactosamine	Influenza virus

For further details, see contents of this review. For more detailed information on the role of CTLs in pathogen recognition, see review [5].
